# Trends in Private Equity Owned Otolaryngology Practice Clinician Distribution

**DOI:** 10.1002/oto2.70112

**Published:** 2025-04-11

**Authors:** Shravan Asthana, Daron Excel, Hemali Shah, Abhinav Talwar, Stephanie Smith

**Affiliations:** ^1^ Department of Otolaryngology–Head and Neck Surgery Feinberg School of Medicine Northwestern University Chicago Illinois USA; ^2^ Department of Otolaryngology–Head and Neck Surgery Medstar Georgetown University Hospital Washington DC USA

**Keywords:** advanced practice providers, audiologists, private equity, private practice, workforce trends

## Abstract

**Objective:**

Investigate how private equity (PE) acquisitions shape the otolaryngology workforce.

**Study Design:**

Cross‐sectional.

**Setting:**

Private outpatient clinics.

**Methods:**

A comprehensive market database, Pitchbook (Seattle, WA), was queried utilizing keywords for outpatient otolaryngology practices acquired by PE firms from 2010 to 2023. Acquisitions were manually verified using practice websites. An open‐source website archive service, WayBack Machine, was utilized to track workforce history of practicing otolaryngologist physicians, audiologists (AuDs), and advanced practice providers (APPs) at these outpatient practices and then subjected to linear regression and Pearson correlation test analysis.

**Results:**

In total, 25 otolaryngology practices were determined to have been acquired by PE, all between 2018 and 2023. Acquisitions predominantly occurred in 2020 (n = 8, 32%) and 2023 (n = 7, 28%) and were mostly in the South Atlantic (n = 10, 40%) or Midwest (n = 8, 32%) regions. From 2018 to 2023, total practice locations increased by 32.0% (n = 194 to n = 256), and the total clinician pool increased by 17.9% (n = 901 to n = 1007). Although physicians (n = 500 to n = 517, *r* = 0.59, *P* = .213) and AuDs (n = 229 to n = 242, *r* = 0.34, *P* = .507) increased nonlinearly, the increase in APPs was strongly linear (n = 172 to n = 248, *r* = 0.95, *P* = .003). As a proportion of the total clinician pool, there was a relative decrease in physicians (55.5%‐51.3%) and AuDs (25.4%‐24.0%), offset by a relative increase in APPs (19.1%‐24.6%).

**Conclusion:**

Our findings indicate a disproportionate increase in APPs relative to physicians and AuDs, although without a comparison group we are unable to draw conclusions in relation to changes in the non‐PE workforce as a whole.

Private equity (PE), which encompasses capital investments made in nonpublicly traded businesses for ownership share, has increasingly invested in private medical practices with the goal of consolidation, improving economies of scale, and negotiating enhanced reimbursements from payors.^1^ Specialties, such as orthopedics, dermatology, ophthalmology, and more recently otolaryngology have become attractive to PE due to their high revenue generation from high surgical and procedural service volume.^1^, ^2^, ^3^, ^4^, ^5^ However, the emergence of PE in medicine has prompted great controversy. Many are concerned that a for‐profit business management strategy may jeopardize patient safety and quality of care, whereas the benefits of PE increase negotiating power with insurers and may enable more streamlined service delivery lines.^6^, ^7^, ^8^ Literature thus far shows that costs increase mostly for patients and payers and has found increases in hospital adverse events and poorer patient outcomes; however, the volume of studies are too low and of mixed results for definitive conclusions about the quality implications.^9^, ^10^, ^11^, ^12^

Otolaryngology is a field with a market described as fragmented, with many small practices in urban areas and high demand for services, making it ripe for PE attention and investment.^13^ As described by Shah et al, PE investment in otolaryngology private practices jumped from one acquisition in 2015 to eight in 2021, with a regional predilection toward the southeastern Atlantic region.^14^ An interesting “secret shopper” study by Haleem et al found that PE‐owned clinics were more likely to accept Medicaid relative to non‐PE clinics (49% vs 29%) although the mean appointment costs were around $90.00 higher (*P* = .004) in PE clinics in the setting of sudden sensorineural hearing loss care.^15^ Other than these two studies, there remains a dearth of research on how PE has impacted otolaryngology practice operations, and there have been no studies to date on its impact on the otolaryngology workforce. Because PE has been investing in specialties such as dermatology and ophthalmology for a greater period of time, the body of literature assessing the impact of PE on patient care and practice patterns in these specialties is more developed. For example, data from dermatology practices acquired by PE indicate that PE acquisition has been associated with an increase in the number of advanced practice providers (APPs), including physician associates (PAs) and nurse practitioners (NPs) at these private dermatology practices.^16^, ^17^ For example, in addition to numerous patient volume and spending differences, Braun et al found that compared to the number of APPs in 2012, 2017 features a 153% increase in PE‐owned dermatology practices compared to 106% increase in non‐PE‐owned counterparts.^16^

This study aims to assess trends between PE acquisitions of otolaryngology practices and changes in the workforce composition of physicians, APPs, and audiologists (AuDs).

## Methods

This study was deemed exempt by the Northwestern University Institutional Review Board. PE deals for otolaryngology and its subspecialties, including head and neck surgery, sleep medicine, pediatric otolaryngology, laryngology, rhinology, facial plastic surgery, and neurotology, were queried from 2010 to 2023 using a comprehensive keyword list previously defined by Shah et al (Supplemental Table S1, available online).^14^ These keywords were entered into Pitchbook (Seattle, WA), a comprehensive market database and repository of publicly available information on both companies and investors. Three rounds of screening were applied to include practices in the study population. First, after querying the full keyword‐based search, filters were applied to select for only the following Pitchbook primary industry codes: clinics/outpatient services, other health care services, hospitals/inpatient services. Then, each practice was subjected to a review of available Pitchbook data to review if the practice offered otolaryngology services or not. Finally, manual validation was applied by searching each practice's website to verify that physicians, AuDs, and APPs were employed at a private practice that offered otolaryngology services, with the primary interlopers being allergy, primary hearing aid, or other device clinics, and ambulatory surgery centers.

After defining the study sample, data on the type of acquisition, parent company, headquartered state, and number of PE deals were extracted from Pitchbook. Geographic locations were defined according to US Census Bureau designations. Meanwhile, data on the staffing and number of locations at each practice were obtained manually using the WayBack Machine, an open‐source internet archive service to manually count the provider listings from practice websites using archived webpages from 2010 to 2023. These data were then subjected to linear regression and Pearson correlation test analyses.

## Results

A total of 621 deals across 556 companies were initially identified using the keyword list. After the application of filters, 177 practices remained. Manual review of available Pitchbook data identified that otolaryngologic services were offered in 100 practices. Finally, manual website review resulted in 25 otolaryngology practices meeting inclusion criteria all in the years 2018 to 2023.

### Acquisition Characteristics

Practices were heavily geographically skewed, with the majority being based in the South Atlantic (n = 10, 40%) and the Midwest (n = 8, 32%). Broken down by state, Texas (n = 6), Florida (n = 5), Illinois (n = 3), Georgia, and Indiana (n = 2) had the most headquartered practices, followed by one each in Minnesota, Wisconsin, North Carolina, Delaware, South Carolina, Michigan, and Louisiana. The most popular year for practices' first PE deal was 2020 (n = 8, 32%) followed by 2023 (n = 7, 28%). On average practices experience 1.6 PE deals, indicating that often more than one deal was made during the investment process. The median age at the first PE deal was 20 years (interquartile range 16‐33) since the practice founding. PE ownership status was classified by Pitchbook as an operating subsidiary in 16 of the practices (64%), whereas privately held in 6 (24%) and simply acquired/merged in 3 (12%). Consequently, buyouts were most common amongst the first deals (n = 20, 80%) with the remaining five practices being invested in through other debt driven vehicles. Finally, most private practices were acquired by common parent companies, with 15 practices being split amongst 5 parent companies and the remaining 10 practices owned by individual PE firms.

### Workforce Trends

**Figure 1:**
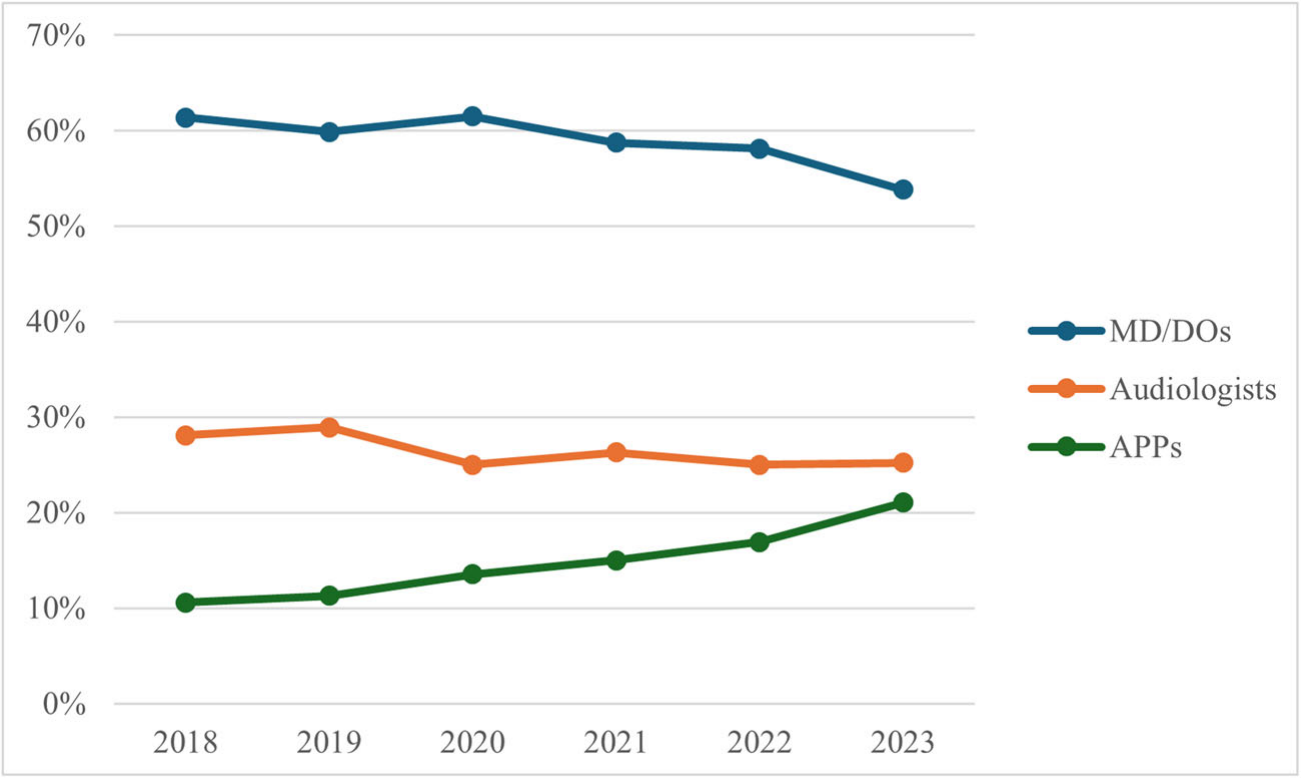
Temporal distribution of total physicians (MD or DO), advanced practice providers (APPs), and audiologists, from 2018 to 2023.

In 2018, 815 clinical staff were employed across the 25 practices included in this study. By 2023, the number of employed clinical staff grew to 961, equating to a 17.9% increase. The number of practice locations also increased by 31.9% (n = 194‐256). When analyzed changes across physicians, APPs, and AuDs, the increase in clinician staff was not distributed equally. As a proportion of the total clinical staff pool from 2018 to 2023, there was a relative decrease in physicians (55.5%‐51.3%) and AuDs (25.4%‐24.0%) offset by a relative increase in APPs (19.1%‐24.6%) (Figure 1). However, although physicians (n = 500 to n = 517, *r* = 0.59, *P* = .213) and AuDs (n = 229 to n = 242, *r* = 0.34, *P* = .507) increased in raw numbers nonlinearly, the increase in APPs over time demonstrates a strong linear relationship (n = 172 to n = 248, *r* = 0.95, *P* = .003) (Table 1). These data are further tabulated by DO, MD, NP, and PA status in Table 2.

**Table 1:** Linear Regression and Pearson Correlation Test Results

Clinician	Correlation coefficient	Pearson's coefficient	*P*‐value	*R* ^2^
MD	4.40	0.33	.526	0.11
DO	4.80	0.77	.070	0.60
PA	15.74	0.98	**<.001**	0.97
NP	7.48	0.83	**.040**	0.69
Physicians	9.20	0.59	.214	0.35
APPs	17.00	0.95	**.003**	0.91
AuD	3.17	0.34	.507	0.11
Locations	14.28	0.96	**.002**	0.93

*Note*: Bold values are statistically significance.

Abbreviations: APP, advanced practice provider; AuD, audiologist; NP, nurse practitioner; PA, physician associate.

**Table 2:** Yearly Clinician Counts From 2018 to 2023^a^

	2018	2019	2020	2021	2022	2023
MD	472 (57.9)	451 (56.4)	452 (58.2)	460 (55.2)	518 (54.4)	461 (48)
DO	28 (3.4)	27 (3.4)	25 (3.2)	29 (3.5)	35 (3.7)	56 (5.8)
Physicians	500 (61.3)	478 (59.8)	477 (61.5)	489 (58.7)	553 (58.1)	517 (53.8)
PA	66 (8.1)	70 (8.8)	88 (11.3)	105 (12.6)	118 (12.4)	144 (15)
NP	20 (2.5)	20 (2.5)	17 (2.2)	20 (2.4)	43 (4.5)	58 (6)
APPs	172 (10.6)	170 (11.3)	179 (13.5)	202 (15.0)	238 (16.9)	248 (21.0)
Audiologists	229 (28.1)	231 (28.9)	194 (25)	219 (26.3)	238 (25)	242 (25.2)
Total clinical staff	815	799	776	833	952	961

Abbreviations: APP, advanced practice provider; NP, nurse practitioner; PA, physician associate.

^a^ Number in parentheses represents the clinician proportion relative to the total clinical staff.

## Discussion

This study aimed to identify how the otolaryngology workforce has changed over time when acquired by PE. Our findings reveal a nearly 50% increase in the number of APPs employed by PE‐acquired otolaryngology practices from 2018 to 2023 along with a linear growth pattern, whereas the number of physicians and AuDs actually decreased as a proportion of all clinical staff in the study population despite increasing in raw numbers over the study period. We also found that the number of practices increased by 31.9% over the study period. Interestingly, our work found that the majority of PE‐acquired otolaryngology practices were concentrated in the South and Midwest, corroborated by the work of Shah et al which may be attributed to many factors not limited to local otolaryngology market dynamics, age of private practices, and individual state regulations.^14^

The notable finding that APPs increased disproportionately in the PE‐acquired otolaryngology workforce may be attributed to several factors, though the reasons behind this are beyond the scope of this investigation. We speculate that several factors contribute to the observed increase in APPs providing otolaryngology care in PE‐owned practices. For one, the increasing demand for otolaryngology services alongside a care shortage, may necessitate the inclusion of APPs to bridge the gap and maintain service delivery.^18^, ^19^ Liu et al have previously demonstrated the crucial role of APPs in the provision of rural ENT care, especially in some counties without an otolaryngologist.^20^ However, even allowing for APP provision of lower intensity services, it is expected that the shortage of otolaryngology providers will continue without larger changes in the training and recruitment structure of the specialty.^21^ Second, APPs are trained more quickly and reimbursed less than otolaryngologists, and therefore there is a decreased cost in employing APPs that may appeal to quickly growing, financially optimized PE‐owned practices.^22^, ^23^ Simultaneously, there are data to support that APPs, and particularly PAs are increasingly shifting toward subspecialty care such as otolaryngology.^24^ Similarly to otolaryngologists, the supply of AuDs is projected to grow to meet increased demand for hearing services.^25^ The decrease observed in this study's relative proportion of AuDs in the total clinician pool could be due to being outpaced by increases in either physicians or APPs, although there may be other audiology‐specific reimbursement and staffing implications involved as described by Wince et al.^26^ Further research may elucidate if these workforce trends are associated with changes in costs, care quality, and access to otolaryngologic care. Additionally, regional health care market dynamics, such as payer mix, regulatory environment, and patient demographics, can drive PE firms’ strategic decisions and should be further explored.

The consolidation of private practices brought about by PE are not in isolation; health care consolidation is simultaneously occurring at a hospital system level such that more physicians, including otolaryngologists, are increasingly becoming employees rather than independently practicing.^27^, ^28^ Moreover, between 2003 and 2017, 282 unique hospitals across 36 states were acquired by PE.^29^ Additionally, according to the 2023 otolaryngology workforce report released by the American Academy of Otolaryngology–Head and Neck Surgery, solo practices are seeing a decline in younger otolaryngologists, whereas academic and nonacademic practices are all increasingly employing APPs. Interestingly, the life cycle of PE investments is known to incentivize more senior attendings in their exit due to the benefit of capital investment, whereas more junior attendings face a rapid ownership change, estimated at 3 to 8 years between parent firms.^30^, ^31^ Thus, otolaryngologists should be aware of how PE might impact their practice upon acquisition.

This study has several limitations. Given that eight practices were acquired in 2023, this prevented a sufficient sample size and time period to assess changes in workforce trends before and after acquisition. Furthermore, the WayBack Machine interface is limited in the number of preceding years it can access, and the selected practices were limited to those identified through Pitchbook. Importantly, this study did not control for non‐PE‐owned practice's workforce changes, which would better isolate acquisition by PE as the factor under study. As a result, the analyses in this study are presented as correlational trends, and further research should estimate the discrete impact of acquisitions with at least 3 years of data before and after the investment as well as compare to academic or non‐PE‐owned private outpatient practices. Another limitation is the lack of data on RNs and MAs, who are key clinical staff members and often increase as practice size increases. Other administrative data on operations or even utilization of care at these practices were unavailable. Finally, although the study period covered 2010 to 2023, the eligibility criteria applied such that only practices acquired from 2018 to 2023 were ultimately included. As acquisitions have occurred over a longer period and continue to occur, future studies with broader eligibility criteria or alternative data sources are warranted.

Our study provides a baseline for how the composition of the otolaryngology workforce is impacted by PE acquisition. Future research should continue evaluating patient, physician, and APP satisfaction, as well as the quality and costs of care provided by otolaryngology practices acquired by PE.

## Conclusion

This study reveals a shift in the workforce composition of otolaryngology practices acquired by PE from 2018 to 2023, with a marked increase in the number of APPs in PE‐acquired practices, and with the acquisitions all occurring in the last 5 years of the study period. This trend may be related to the strategic role of APPs in bridging care gaps amid increasing demand and physician shortages, as well as the cost‐efficiency that PE firms may be seeking. The growth in the number of practice locations and total clinical staff also suggests that PE acquisitions may drive expansion and consolidation in the otolaryngology sector. However, the potential implications for care quality and costs necessitate further investigation. Without a comparison group, we are unable to draw conclusions in relation to changes in the non‐PE workforce as a whole. As PE investment and, more broadly, health care consolidation continue, understanding the impact of PE on otolaryngology care quality and costs is crucial for informing future workforce strategies, policy decisions, and care delivery.

## Author Contributions

**Shravan Asthana**, design, data acquisition, statistical analysis, data interpretation, drafting, and revision; **Daron Excel**, data acquisition, data interpretation, drafting, and revision; **Hemali Shah**, design, data acquisition, data interpretation, and revision; **Abhinav Talwar**, design, statistical analysis, data interpretation, and revision; **Stephanie Smith**, design, data acquisition, statistical analysis, data interpretation, revision, and supervision.

## Disclosures

### Competing interests

The authors declare no conflicts of interest.

### Funding source

The authors received no financial support for authorship and/or publication of this article. Dr. Smith is supported by National Institute of Allergy and Infectious Disease grants 5P01AI145818 and R01AI175631.

## Supporting information


**Supplemental Table 1:** Keyword list entered into Pitchbook to identify private equity investments in otolaryngology adapted from Shah et al (2023).^14^
