# Supplementary material for: Trends in Private Equity Owned Otolaryngology Practice Clinician Distribution
Source: OTO Open. 2025 Apr 11;9(2):e70112. doi: 10.1002/oto2.70112 (PMC11986684; doi:10.1002/oto2.70112)
Supplement: Supplementary file 1 — Supplemental Table 1: Keyword list entered into Pitchbook to identify private equity investments in otolaryngology adapted from Shah et al (2023).14 [file OTO2-9-e70112-s001.docx]

| Otolaryngology  Otorhinolaryngology  Ear Nose Throat  Laryngology  Otology  Rhinology  Facial Plastics  Allergy  Sinus  Audiology  Asthma  Facial reconstruction  Vestibular disorders  Head and neck surgery  Hearing loss  Otogenic  Pharyngology  Cranial  Thyroid  Parathyroid  Skull base  ENTA  Allergy, Asthma, and Immunology  Voice and Swallowing  Multispecialty  Ambulatory ENT  Outpatient ENT  Inpatient ENT  Nasal  Paranasal  Auricular  Vocal  Salivary  Tonsillar  Facial plastic  Facial reconstructive  Oto  Cosmetic surgery  Voice  Swallow |
| --- |
